# Diagnostic imaging for spinal disorders in the elderly: a narrative review

**DOI:** 10.1186/2045-709X-20-16

**Published:** 2012-05-24

**Authors:** John AM Taylor, André Bussières

**Affiliations:** 1 Department of Chiropractic, D’Youville College, 320 Porter Avenue, Buffalo, NY, 14201, USA; 2 Département chiropratique, Université du Québec à Trois-Rivières, 3351, boul. Des Forges, C. P. 500, Trois-Rivières (Québec), G9A 5H7, Canada; 3 PhD Population Health program, University of Ottawa, Ottawa, Canada

**Keywords:** Narrative review, Low back pain, Neck pain, Diagnostic imaging, Radiography, Computed tomography, Magnetic resonance imaging, Aging, Geriatric

## Abstract

The high prevalence of neck and low back pain in the rapidly aging population is associated with significant increases in health care expenditure. While spinal imaging can be useful to identify less common causes of neck and back pain, overuse and misuse of imaging services has been widely reported. This narrative review aims to provide primary care providers with an overview of available imaging studies with associated potential benefits, adverse effects, and costs for the evaluation of neck and back pain disorders in the elderly population. While the prevalence of arthritis and degenerative disc disease increase with age, fracture, infection, and tumor remain uncommon. Prevalence of other conditions such as spinal stenosis and abdominal aortic aneurysm (AAA) also increase with age and demand special considerations. Radiography of the lumbar spine is not recommended for the early management of non-specific low back pain in adults under the age of 65. Aside from conventional radiography for suspected fracture or arthritis, magnetic resonance imaging (MRI) and computed tomography (CT) offer better characterization of most musculoskeletal diseases. If available, MRI is usually preferred over CT because it involves less radiation exposure and has better soft-tissue visualization. Use of subspecialty radiologists to interpret diagnostic imaging studies is recommended.

## Background

According to the United States Census Bureau, between the years 2000 and 2010, the United States (US) population aged 45 to 64 years and 65 years and over grew at rates of 31.5 percent and 15.1 respectively [1]. This rapid growth in the aged population is a consequence of an increase in life expectancy as well as the “Baby Boom” generation. Every year over 3.5 million baby boomers in the US turn 55 leading to predictions that by 2035, 20 percent of the population will be 65 or older [2]. A similar trend is observed in Australia where the proportion of the population aged 65 years and over increased from 11.1 % to 13.5 % between the years 1990 and 2010 [3]. Unsurprisingly, this aging trend is associated with an increased use of health care services for a number of heath conditions.

For instance, neck pain (NP) and low back pain (LBP) are common complaints in seniors, leading to impaired functional ability and decreased independence. A recent cross sectional study suggests between 10-20 % of seniors over the age 70 reported more than 30 days of NP or LBP within the past year, with a significant proportion having diminished their physical activities due to NP (11 %) or LBP (15 %) within the past year [4]. Poor overall physical function, bad self-rated health, and higher depression scores have been associated with higher prevalence of neck and back pain [5]. Most neck and back pain sufferers seeking help consult primary care professionals, including chiropractors [6,7].

Chiropractors specialize in managing musculoskeletal disorders and reviews have indicated that this approach is as effective and safe as conventional medical care and physiotherapy for back pain [8-10]. When combined with other modalities such as exercise, chiropractic care appears to be more effective than other treatment approaches for patients with chronic neck pain [9,11,12]. For acute and subacute neck pain, cervical manipulation appears to be more effective than medication in both the short and long term. However, a few instructional sessions of home exercise resulted in similar outcomes [13].

However, knowledge practice gaps have been reported among various primary care practitioner groups in the assessment of red flags and the use of diagnostic imaging [14,15]. Furthermore, evidence of overuse and misuse of imaging services for spine disorders has been reported in the medical [16-19] and chiropractic literature [15,20-23].

In practical terms, overuse of imaging results in unnecessary tests or procedures with associated risks and side effects [24], two issues that are of significance both at the clinical and the population health level. Potential adverse outcomes of overuse of imaging include inefficient and potentially inappropriate invasive diagnosis and subsequent treatment [25,26], unnecessary ionizing radiation exposure [27,28], increased waiting time for treatment, added costs, and poor utilization of human resources [26,29]. Combined with increasing technological advancements, overuse of diagnostic imaging in the aging population presenting with prevalent conditions such as neck and back pain results in significant increases in health care costs and associated adverse outcomes [30]. As a consequence, clinicians need to make informed decisions regarding optimal management and evaluation of the more vulnerable populations.

This review aims to provide primary care providers with an overview of available imaging studies with associated potential benefits, adverse effects, and costs for the evaluation of neck and back pain disorders in the elderly population.

## Specific causes of back and neck pain

Current evidence suggests that most routine radiography of the spine is unnecessary during the initial evaluation of patients with LBP or NP unless specific clinical indicators suggestive of serious underlying conditions (red flags) are present [19,31,32]. In the absence of these red flags, lumbar spine radiography rarely reveals the source of the patient complaint and does not improve clinical outcomes (short and long term quality of life, pain and function, mental health or overall improvement) compared with usual clinical care without immediate imaging [19].

While in about 10 % of cases a specific cause of LBP can be identified, less than half of these have serious underlying pathologies such as cancer, infection, and fractures [33], and the prevalence of these diseases is even lower in neck pain (NP) patients [34-39]. Reviews suggest that the prevalence of fracture (0.7-4 %), possible infection (0.1-0.8 %), and possible tumor (0.3-3.8 %, 0.7 % typically reported) are all quite low [33,40-45]. While fracture and possible infection showed no association with age in a retrospective review of 2000 radiographic studies, possible tumor was reported only in patients over age 55 [46]. The prevalence of lumbar spine degeneration increased with age to 71 % in patients aged 65–74 years. Except for symptomatic degenerative spinal stenosis (3 %) [40,47], therapeutic consequences of detecting degenerative changes are minor [46,48]. The prevalence of an inflammatory disorder (about 1 %) and progressive or painful structural deformity including scoliosis and kyphoscoliosis (less than 1 %) also remains low [49,50]. It is estimated that abdominal aortic aneurysm, a pathology sometimes mimicking LBP, affects up to 8 % of men over 65 years of age, and is becoming increasingly common in women [51].

Cervical spine myelopathy may result from a number of conditions, including trauma, tumors, infection, vascular disease, degenerative conditions, demyelinating disorders, spinal stenosis, and central cervical disc herniation. Atlantoaxial instability should also be suspected in patients with active inflammatory arthritis, congenital disorders and hereditary connective tissues disorders, and traumatic disorders [52].

## Past reviews

In a best-evidence review of diagnostic procedures for neck and low back pain, Rubenstein and van Tulder emphasized that although most spinal conditions are benign and self-limiting, the real challenge to the clinician is to distinguish serious spinal pathology or nerve-root pain from non-specific neck and low-back pain. In their investigation, they identified four systematic reviews that evaluated the diagnostic accuracy of diagnostic imaging for LBP, but could find no such reviews for neck pain [53].

According to one of the reviews, imaging findings of degenerative changes were weakly associated with non-specific LBP, while spondylolysis, spondylolisthesis, spina bifida, transitional vertebrae, spondylosis and Scheuermann disease were not associated with non-specific LBP [54]. It is unclear from the above review, however, whether the authors were referring to acute (active) or chronic spondylolysis (pars interarticularis defect).

Jarvik and Deyo concluded that for patients 50 years of age and older, or those who have red flag findings that suggest systemic disease, conventional radiography with standard laboratory tests can almost completely rule out underlying systemic disease and that CT and MRI should be used only in surgical candidates and patients in whom systemic disease is strongly suspected [55].

With regard to invasive lumbar spine discography, Staal reported that the specificity and sensitivity are high for the diagnosis of disc degeneration. However, the accuracy of discography for the diagnosis of discogenic pain has never been established owing to the lack of an adequate gold standard [56]. Furthermore, discography is rarely performed in older individuals. Similarly, the use of single photon emission computed tomography (SPECT) imaging is not supported by evidence [57], and is used infrequently to evaluate LBP in older individuals.

The accuracy of image interpretation is an important consideration in any discussion of diagnostic imaging. Arriving at a correct diagnosis involves not only the appropriate selection of imaging study to be performed, but also an accurate interpretation of the images once they are obtained. Taylor et al conducted a study comparing students, clinicians, radiology residents and radiologists in the interpretation of abnormal lumbosacral spine radiographs in medicine and chiropractic. The data revealed a substantial increase in the percentage of correct diagnoses in interpretations by radiologists and radiology residents compared to interpretations by chiropractors, medical clinicians, and students. The study reinforced the need for radiologic specialists to reduce missed diagnoses, misdiagnoses, and medicolegal complications [58].

## Clinical guidelines for imaging of spine disorders

The Clinical Guidelines Committee of the American College of Physicians (ACP) concluded that diagnostic imaging is indicated for patients with low back pain only if they have severe progressive neurologic deficits or signs or symptoms that suggest a serious or specific underlying condition. In other patients, evidence indicates that routine imaging is not associated with clinically meaningful benefits but can lead to harms. They concluded that more testing does not equate to better care and that implementing a selective approach to low back imaging, as suggested by the ACP and American Pain Society guideline on low back pain, would provide better care to patients, improve outcomes, and reduce costs [59]. Table 1 offers suggestions for imaging in patients with LBP (alone or with leg pain).

**Table 1:** Suggestions for Imaging in Patients with Low Back Pain (alone or with leg pain)*

**Indicators for Initial Imaging**	**Imaging Action**
	***Immediate imaging***
· Risk factors for cancer (multiple risk factors for cancer, or strong clinical suspicion for cancer)	Radiography plus ESR^†^
· Risk factors for cancer (history of cancer with new onset of LBP)	MRI (or CT if MRI not available)
· Risk factors for spinal infection (new onset of LBP with fever and history of intravenous drug use or recent infection)	
· Risk factors for or signs of the cauda equina syndrome (urine retention, motor deficits at multiple neurologic levels, fecal incontinence, or saddle anesthesia)	
· Severe neurologic deficits (progressive motor weakness)	
	***Defer imaging after a trial of therapy****(about 1 month)*
· Weaker risk factors for cancer (unexplained weight loss or age >50 years)	Radiography with or without ESR
· Risk factors for or signs of ankylosing spondylitis (morning stiffness that improves with exercise, alternating buttock pain, awakening because of back pain during the second part of the night, or younger age [20 to 40 years])	
· Risk factors for vertebral compression fracture (history of osteoporosis, corticosteroid use, significant trauma, or older age [>65 for men or >75 for women])	
· Signs and symptoms of radiculopathy (back pain with leg pain in an L4, L5 or S1 nerve root distribution or positive result on straight leg raise or crossed straight leg raise test) in patients who are candidates for surgery or epidural steroid injection	MRI (or CT if MRI not available)
· Risk factors for or symptoms of spinal stenosis (radiating leg pain, older age, or pseudoclaudication) in patients who are candidates for surgery	
	***No imaging***
· No criteria for immediate imaging and back pain improved or resolved within 1-month trial of therapy	
· Previous spinal imaging with no change in clinical status	

* Adapted from Chou R, Qaseem A, Snow V, Casey D, Cross JT Jr, Shekelle P, et al; Clinical Efficacy Assessment Subcommittee of the American College of Physicians. Diagnosis and treatment of low back pain: a joint clinical practice guideline from the American College of Physicians and the American Pain Society. Ann Intern Med. 2007, 147:478–91. [PMID: 17909209]

† Consider MRI if the initial imaging result is negative but a high degree of clinical suspicion for cancer remains.

In 2007 and 2008 the authors published diagnostic imaging practice guidelines for musculoskeletal complaints in adults of all ages [52,60-62]. For back pain patients in general, a number of “red flags” have been identified that indicate possible underlying systemic or local pathology such as tumor, infection, fracture or inflammatory arthropathy. It is important to understand, however, that some red flags are associated with significantly high false-positive rates, indicating that, when used in isolation, they have little diagnostic value in the primary care setting [63]. In an inception cohort of 1,172 consecutive patients receiving primary care for acute low back pain, age alone as a red flag ‘LBP onset before 20 years or over 55 years’ for possible cancer had a false positive rate of 24.0 % (95 % Confidence Interval: 21.6–26.5). As a result, the 50-year old criterion proposed in many earlier guidelines has been questioned. Some authorities now recommend that this cutoff should apply to patients older than 65 years [64]. Two recent high quality national guidelines have considered the field of manual therapy when making recommendations:

For acute LBP, immediate imaging is recommended in patients who have major risk factors for cancer, risk factors for spinal infection, risk factors for or signs of the cauda equina syndrome, or severe or progressive neurologic deficits. Lumbar radiographs with or without erythrocyte sedimentation rate is recommended after a trial of therapy in patients with: 1) minor risk factors for cancer (unexplained weight loss or age > 50 years) and no neurological deficit; and 2) risk factors for vertebral compression fracture (history of osteoporosis, use of corticosteroids, significant trauma, or older age [> 65 years for men or > 75 years for women]), signs or symptoms of radiculopathy, or risk factors for or symptoms of symptomatic spinal stenosis. Repeat imaging is only recommended in patients with new or changed low back symptoms, such as new or progressive neurologic symptoms or recent trauma [59].

For persistent (less than 12 weeks) non-specific LBP in patients over age of 18, National Institute for Health and Clinical Excellence (NICE) (2009) recommendations are as follows [65]: 1) Do not offer radiography of the lumbar spine for the management of persistent non-specific low back pain; 2) Consider MRI when a diagnosis of spinal malignancy, infection, fracture, cauda equina syndrome or ankylosing spondylitis or another inflammatory disorder is suspected; 3) Only offer an MRI scan for non-specific low back pain within the context of a referral for an opinion on spinal fusion. Further, a combination of red flags significantly increases the likelihood of finding a serious pathology [60]. Clinical decision rules for cervical spine trauma patients also use age 65 or older as one high risk factor that warrants obtaining radiographs [66-68].

A similar ongoing debate concerns the symptom duration necessary to warrant the recommendation of lumbar spine radiographs in LBP. The duration ranges from as low as 4 weeks [52] up to 7 weeks [64] for the patient with a first episode of low back pain who has not been treated or who is not improving with conservative treatment. More recent reviews however suggest that clinicians should refrain from ordering lumbar radiographs for non-specific LBP (i.e. absence of red flag indicators of serious pathology and no severe disabling pain) for acute, subacute and persistent LBP of less than 12 months [59,65]. One question that remains then is: How many weeks of conservative care are appropriate before one proceeds with further investigations? Should it be four weeks or perhaps seven weeks? It may be that the pain is unresponsive to physical and pharmaceutical intervention because it now results from ineffective endogenous pain control and central sensitization in which case imaging studies would be of little help [69]. Obviously, further research is necessary before making a useful recommendation. In any event, a conservative approach to imaging is warranted at this time.

In summary, no imaging is recommended for acute, subacute and persistent LBP in patients under age 65 years unless: 1) spinal malignancy, infection, fracture or inflammatory disorder is suspected; 2) patient is not improved or has significant functional deficits after a trial of therapy of four weeks (low force, low velocity techniques suggested). An initial trial of therapy of four weeks (using low force, low velocity techniques) may be offered in patients with: 1) minor risk factors for cancer (initial imaging can include lumbar radiography and evaluation of erythrocyte sedimentation); and 2) non progressive signs or symptoms of radiculopathy or spinal stenosis. For patient with risk factors for vertebral compression fracture, dual energy x-ray absorptiometry (DXA) is indicated to detect and quantify osteoporosis (see osteoporosis section below). Gentle techniques should be used if an initial trial of therapy is suggested for these patients. Decisions regarding repeated imaging should be based on the development of new or changed clinical features.

## Condition-specific clinical guidelines for imaging of spine disorders *(For Adults in General)*

### Traumatic spine disorders

#### Thoracolumbar, Lumbar and Thoracic Spine Trauma [52]

Radiographs are not routinely indicated in the following settings:

a. recent acute thoracolumbar, lumbar or thoracic spine trauma for less than 2 weeks duration in adult patients with absence of pain, normal range of motion, and absence of neurologic deficits;

b. posttraumatic chest wall pain in patients with minor trauma. Rib fractures are difficult to visualize. Clinical suspicion warrants altering treatment plan in such patients (use low force, low velocity techniques);

c. coccyx trauma and coccydynia but radiographs should be considered if distal sacral fracture is suspected.

Radiographs are indicated in the following settings:

a. recent acute thoracolumbar, lumbar or thoracic spine blunt trauma or acute injuries such as falls, motor vehicle collisions, motorcycle, pedestrian, or cycling injuries;

b. posttraumatic chest wall pain in patients with major trauma.

c. Pelvis and sacrum trauma including falls with inability to bear weight

CT or MRI should also be considered in the above settings. Nuclear medicine (bone) scan may be helpful when radiographs are normal or equivocal for fracture.

Reinus and colleagues studied indications for lumbosacral spine radiographs in 482 patients presenting to a Level II emergency department. The major indications for lumbosacral radiographs were lower back pain (92 %) and trauma (36 %). However, patient expectation and medicolegal concerns, related either to insurance documentation or to physician litigation, were cited in 42 % of cases despite the fact that these are not appropriate indications for imaging. They concluded that their data supported the use of lumbosacral spine radiographs for patients with a history of trauma, even if relatively minor, in elderly patients and in patients with lower back pain who have a history of neoplasm. However, the data revealed that lumbosacral radiographs obtained for an isolated complaint of lower back pain or isolated neurologic abnormalities generally provide no clinically useful information. They concluded that such patients are better examined (although not necessarily at the time of emergency department evaluation) with techniques such as MR imaging that reveal soft-tissue lesions. [70]

#### Cervical spine trauma [52]

**Figure 1:**
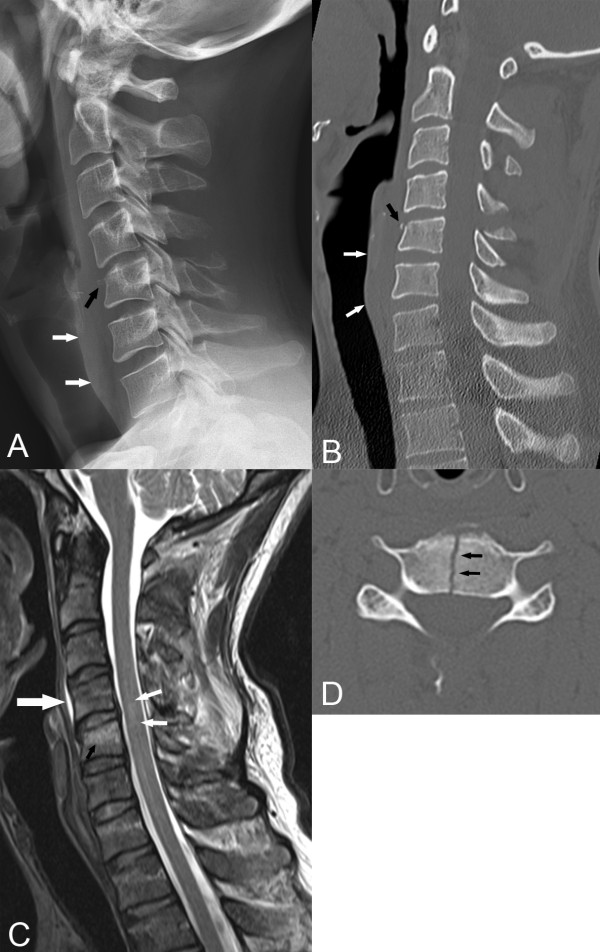
**Cervical Spine Trauma. A.** A lateral cervical spine radiograph reveals a C4 spinous process fracture with inferior displacement. The C5 vertebral body is slightly compressed with a tiny teardrop fragment anteriorly (black arrow). The prevertebral soft tissue margin is clearly visualized and there is a suggestion of widening secondary to edema. (white arrows) **B.** A sagittal reconstruction of a CT scan reveals the same findings as the radiograph but in much more detail. **C.** A sagittal T2-weighted MR image reveals high signal intensity in the C5 vertebral body (black arrow), in the posterior soft tissues, and within the cord itself. The latter finding is characteristic of spinal cord contusion, edema, and/or hemorrhage. **D.** An axial CT image shows a complete vertical fracture (arrows) through the C5 vertebral body, a finding not seen on the lateral radiograph or sagittal CT display. (Images courtesy of Lindsay J. Rowe, Newcastle, Australia).

In alert and stable cervical spine trauma patients, radiographs are only routinely indicated in patients with positive high-risk factors on the Canadian Cervical Spine Rule for Radiography in Alert and Stable Trauma Patients (CCSR) [66,71]. One of those factors is age over 65. Therefore, all patients over age 65 should get a 3-view routine cervical spine radiographic series (anterior-posterior, lateral, and anterior-posterior open mouth), in acute cervical spine trauma. If fracture is suspected, CT is recommended rather than oblique, pillar or flexion-extension radiographs. MRI may also be indicated in certain cases to evaluate soft tissue, cord or nerve root injury. (Figure 1)

The Bone and Joint Decade 2000–2010 task force on neck pain and its associated disorders (TFNP) concluded that CT scans have better validity (in adults and elderly) than radiographs in assessing high-risk and/or multi-injured blunt trauma neck patients. There is no evidence, on the other hand, that specific MRI findings are associated with neck pain, cervicogenic headache, or whiplash exposure. Furthermore, flexion-extension radiographs and 5-view radiographs (cross table lateral, anterior-posterior, bilateral oblique, and odontoid views) in the acute stage of blunt neck trauma add little to static radiography in predictability and accuracy [31].

### Uncomplicated spine disorders

(Defined as nontraumatic mechanical pain that varies with time and activity with no neurologic component and a good general health status).

#### Uncomplicated Thoracic and Lumbar Spine Disorders [52]

Conventional radiographs are not initially indicated in adult patients with acute, subacute, or persistent uncomplicated LBP with no neurologic deficits or red flags. As a general rule, a 4–6 week therapeutic trial of conservative care is appropriate before radiographs are obtained. However, since age 65 or over is considered a red flag, radiographs are often indicated at the time of initial presentation, especially if the patient has at least one additional red flag. Additionally, lumbar spine radiographs are indicated in patients over 65 or those who have progressive neurologic deficits with suspected degenerative spondylolisthesis, lateral stenosis, or central stenosis. Oblique or flexion-extension radiographs, CT or MRI are not initially indicated in these patients and should be reserved for those with a failed 4–6 week trial of conservative care or deteriorating neurologic deficit or disabling leg pain.

#### Degenerative lumbar spinal stenosis (DLSS)

According to the North American Spine Society (NASS) evidence-based clinical guideline for the diagnosis and treatment of DLSS, MRI is the most appropriate, noninvasive test for imaging degenerative lumbar spinal stenosis. These guidelines further recommend that CT myelography is useful in patients who have contraindications to MRI, patients with MRI findings that are inconclusive, or patients with a poor correlation between symptoms and MRI findings. CT without myelography is useful in patients who have contraindications to MRI, patients with MRI findings that are inconclusive, or patients with a poor correlation between symptoms and MRI findings and those who are not candidates for CT myelography. [72]

#### Degenerative lumbar spondylolisthesis (DLS)

**Figure 2:**
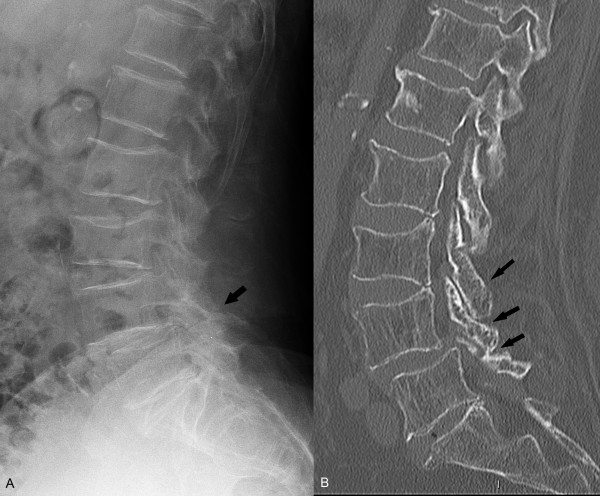
**Degenerative Lumbar Spondylolisthesis (DLS).** A lateral radiograph **(A)** and sagittal reconstructed CT image **(B)** of this 78 year old woman shows a 10 % anterolisthesis of L4 associated with severe facet joint osteoarthrosis (arrows). In this patient, severe osteoporosis has led to a fragility fracture of the L3 vertebral body. (Images courtesy of Lindsay J. Rowe, Newcastle, Australia).

According to the Evidence-Based Clinical Guideline developed by the DLS Work Group of NASS, the most appropriate, noninvasive test for detecting DLS is the lateral radiograph, whereas the most appropriate, noninvasive test for imaging the stenosis associated with DLS is MRI. (Figure 2) As in imaging recommendations for DLSS, plain myelography or CT myelography are also useful for assessing spinal stenosis associated with DLS. CT without myelography is a useful noninvasive study in patients who have contraindications to MRI, patients with MRI findings that are inconclusive, or patients with a poor correlation between symptoms and MRI findings and those who are not candidates for CT myelography [73].

#### Intervertebral disc disorders

Conventional radiographs are not initially indicated in suspected acute lumbar disc herniation (protrusion, extrusion, sequestration) unless the patient is over age 50 or has progressive neurologic deficits. However, radiographs are insensitive to disc herniations and acute disc herniations occur mostly in the 35–54 year age range. While degenerative disc bulges are more likely to occur in older individuals, they are not visible on radiographs either [52].

**Figure 3:**
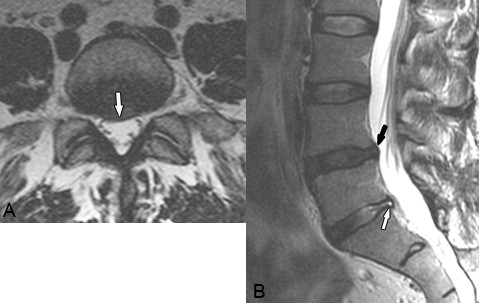
**Disc Bulge. A.** Axial and **B.** Sagittal T2-weighted images reveal a focal right-central disc bulge at L4-5 that slightly indents the thecal sac and extends into the right nerve root canal (white arrow on A, black arrow on B). A more focal protrusion and associated annular tear is present at L5-S1 (white arrow on B). While degenerative changes such as disc bulges are extremely prevalent, the only degenerative feature associated with LBP is spinal stenosis. While disc herniations such as protrusions, extrusions, and sequestrations are more likely to result in direct nerve compression and chemical radiculitis resulting in lower extremity symptoms, they occur less frequently in elderly patients. (Images courtesy of Brian A. Howard, Charlotte, NC).

One of the difficulties in evaluating the utility and validity of MRI in LBP is the high prevalence of abnormal findings in asymptomatic individuals. A recent systematic review and meta-analysis by Endean et al concluded that MRI findings of disc protrusion, nerve root displacement or compression, disc degeneration, and high intensity zone are all associated with LBP, but that individually, none of these abnormalities provides a strong indication that LBP is attributable to underlying pathology [74]. (Figure 3) This limits the value of abnormal MR imaging findings in evaluating intervertebral disc disorders and degenerative changes in elderly patients with LBP.

Kalichman et al retrospectively evaluated spinal degeneration in a subset of 187 participants with a mean age of 52.6 years of age who initially underwent multidetector CT scans primarily to assess aortic calcifications. While degenerative changes were extremely prevalent, the only degenerative feature associated with self-reported LBP was spinal stenosis. Intervertebral disc space narrowing (present in 63.9 % of spines), and facet joint osteoarthrosis (64.5 %) were unassociated with LBP [48].

#### Cervical spine disorders [52]

Conventional radiographs or special investigations are not initially indicated in uncomplicated (no neurologic deficits or red flags), nontraumatic neck pain of less than four weeks duration. Radiographs are indicated, however, for patients with nontraumatic neck pain and radicular symptoms. This category includes patients with suspected acute cervical disc herniation or suspected acute cervical spondylotic radiculopathy or lateral canal stenosis. While the three-view series of radiographs are suggested, oblique or swimmer (spot lateral cervicothoracic) views may also be included. Cervical spine MRI should be considered after a failed four-week trial of conservative therapy.

The TFNP recommends that radiographs are not even initially indicated in patients with uncomplicated subacute (4–12 week duration) and persistent (>12 week duration) neck pain with or without associated arm pain. They recommend a system of “Red Flags” (similar to those now used in assessing patients with low back pain), that allow clinicians to rule out serious pathology in patients seeking care for neck pain with no exposure to blunt trauma. (Table 2) Important serious disorders to consider include pathologic fractures, neoplasm, systemic inflammatory disease, infection, cervical myelopathy, and/or previous cervical spine or neck surgery or open injury [31].

**Table 2:** Suggested “Red Flags” for Triage of Patients Seeking Nonemergency Care for Neck Pain*

Suggested "Red Flags"	Definition
Trauma: Suspected Fragility Fracture	Minor or no trauma but decreased bone mass due to osteoporosis or corticosteroid therapy
Tumor/Cancer/Malignancy	Previous history of cancer, unexplained weight loss, failure to improve with a month of therapy
Spinal Cord Compromise	Cervical myelopathy (where about half of patients with cervical myelopathy have pain in their neck or arms; most have symptoms of arm, leg or, uncommonly, bowel and bladder dysfunction)
Systemic Diseases	Ankylosing spondylitis or other inflammatory arthritis
Infections	Intravenous drug abuse, urinary tract infection, or skin infection
Pain	Intractable pain, tenderness over vertebral body
Prior Medical History	Previous neck surgery

* Adapted from Nordin M et al. **Assessment of neck pain and its associated disorders: results of the Bone and Joint Decade 2000–2010 Task Force on Neck Pain and Its Associated Disorders**. Spine 2008,**15**;33(4 Suppl):S101-22.

### Complicated spine disorders

(Defined as the presence of red flag clinical indicator(s) that should alert the clinician to possible underlying pathology)

### Thoracic and lumbar spine pain

Advanced imaging including MRI, CT or nuclear medicine (NM) bone scan are recommended in all adult patients with complicated thoracic or lumbar spine pain with red flags and indicators of contraindication to SMT [52].

#### Cauda equina syndrome (CES)

Elderly patients with CES (presenting as LBP, bilateral or unilateral sciatica, saddle anesthesia, motor weakness of the lower extremities that may progress to paraplegia, urinary retention, or bowel and/or bladder incontinence) should be treated as a surgical emergency requiring immediate emergency referral. There is no value in obtaining imaging prior to the referral as the imaging studies will likely be repeated at the emergency facility [65].

#### Abdominal aortic aneurysm (AAA)

**Figure 4:**
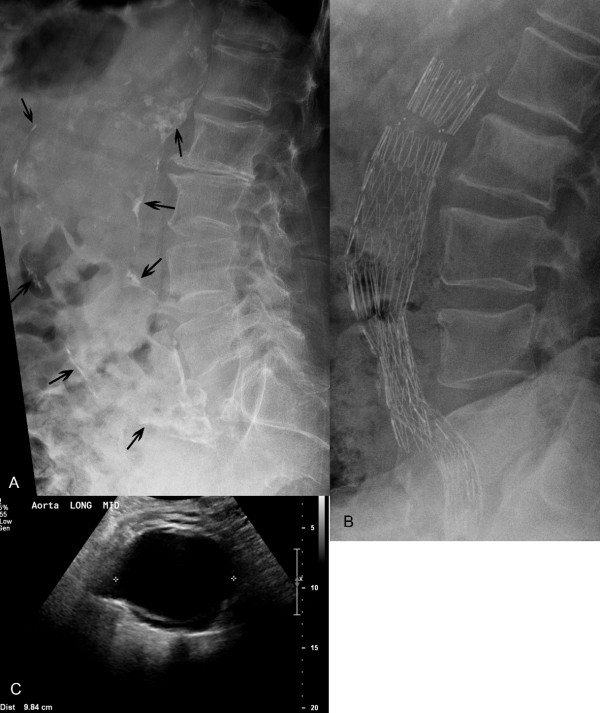
**Abdominal Aortic Aneurysm. A.** In this 68 year old man, a lateral radiograph reveals severe atherosclerotic plaques depicted as conduit wall calcification outlining a 9 cm diameter aneurysm (arrows) extending from the upper abdominal aorta to the iliac arteries near the lumbosacral junction. **B.** In another patient, observe the metallic mesh of an aortic and iliac artery graft. This patient also has skeletal metastasis with osteoblastic lesions within the L2 and L5 vertebral bodies. **C.** A longitudinal diagnostic ultrasound image through the center of an aneurysm (same patient as in A) documents that the diameter of the lumen at its maximum width is 9.84 cm. (Images courtesy of Lindsay J. Rowe, Newcastle, Australia).

AAA is a vascular disease with life-threatening implications that affects about 4-9 % of men and 1 % of women, mostly age 65 and over. AAA commonly presents as back pain and therefore may be encountered in elderly patients seeking chiropractic care. In non dissecting AAAs, medical referral and ultrasound are recommended even if conventional radiographs are negative (calcification, the most reliable radiological sign, is seen in only 50 % of AAA) [75]. In 2005 the US Preventive Services Task Force (USPSTF) published a recommendation that all men between the ages of 65 and 75 who are or have been smokers should have a one-time abdominal diagnostic ultrasound study (DUS) to screen for AAA. They emphasized that 70 percent of men in this age group have smoked and would benefit from routine screening to check for aneurysms. The USPSTF make no recommendation about AAA screening for men between the ages of 65 and 75 who have never been smokers and they recommend against such routine DUS screening for AAA in women [76]. In the US, Medicare covers the cost of this one-time screening DUS in patients with a family history of AAA or who have smoked at least 100 cigarettes in their lifetime [77]. (Figure 4)

Suspected acute AAA or thoracic aortic aneurysm, dissection, rupture, occlusion or traumatic injury in any patient requires immediate emergency referral without imaging [52].

#### Osteoporosis

Conventional radiographs are notoriously unreliable for assessing bone mineral density (BMD). In elderly patients with or without fragility fractures, dual energy x-ray absorptiometry (DXA) is indicated to detect and quantify osteoporosis. The decision to test BMD should be based on a woman's clinical risk profile, as well as the potential impact of results on management [78]. Regardless of clinical factors, all women over age 65 and all males over age 70 should be tested for BMD. BMD testing is also recommended for postmenopausal women younger than 65 with osteoporotic risk factors and in men aged 50–69 if at least one major or two minor risk factors for osteoporosis are present [78]. Several of these important osteoporosis risk factors have been identified that place elderly patients, especially postmenopausal females, at risk. (Table 3) The FRAX® tool was developed by the World Health Organization to evaluate fracture risk in both postmenopausal women and men aged 40 to 90 years. It is validated to be used in untreated patients only. The current National Osteoporosis Foundation Guide is based on individual patient models that integrate the risks associated with clinical risk factors as well as BMD at the femoral neck. The FRAX® algorithms give the 10-year probability of fracture of the spine, forearm, femoral neck, or proximal humerus [79]. Simplified paper versions, based on the number of risk factors can be downloaded for office use at: http://www.shef.ac.uk/FRAX/. For most people, an interval of at least two years is an appropriate duration for repeating BMD testing.

**Table 3:** Important Osteoporosis Risk Factors [104,105]

**Major risk factors**	**Minor risk factors**
Vertebral compression fracture	Rheumatoid arthritis
Fragility fracture after age 40	Past history of hyperthyroidism
Family history of osteoporotic fracture	Chronic anticonvulsant therapy
Systemic glucocorticoids >3 months	Low dietary calcium intake
Malabsorption syndrome	Smoking
Primary hyperparathyroidism	Excessive alcohol intake
Propensity to fall	Excessive caffeine intake
Osteopenia apparent on x-ray film	Weight <57 kg
Hypogonadism	Weight loss >10 % of weight at age 25
Early menopause (before age 45)	Long term heparin therapy

**Figure 5:**
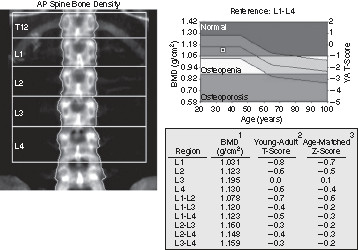
**Dual Energy X-ray Absorptiometry (DXA) for Osteoporosis.** This DXA display printout shows the results of a typical normal study. DXA scans are used to quantify bone mass that is expressed in standard deviations from the normal. This information can be applied to management, prognosis, and estimation of fracture risk in patients with osteopenia or osteoporosis. DXA is the most common study used as a screening test in women over age 65 and others at risk for osteoporosis. (Reprinted with permission from Taylor JAM, et al, Skeletal Imaging, Atlas of the Spine and Extremities. 2010, 2^nd^ edition. St. Louis, Elsevier.).

In the US, Medicare covers the cost of DXA scans once every 24 months to determine fracture risk in people who are at risk for osteoporosis [80]. In Australia, Medicare has covered bone mineral density tests for all patients aged 70 years and over since April 2007 [81]. (Figure 5)

#### Compression fracture

Conventional radiographs are indicated for the initial evaluation of suspected thoracic and lumbar spine compression fractures. Additional MRI or CT evaluation is indicated in cases where initial radiographs are positive, difficult to interpret, or when complex lesions or ligamentous instability or neural injuries are suspected. (Figure 2) MRI is also useful in determining whether fractures are acute or chronic and also prior to kyphoplasty procedures, for surgical planning, and to detect incidental pathology [82]. Fluorodeoxyglucose positron emission tomography fused with computed tomography (FDG-PET/CT) is useful in differentiating benign from malignant compression fractures [83]. The use of PET/CT is limited, however, by its considerable expense.

#### Plasma cell (multiple) myeloma

**Figure 6:**
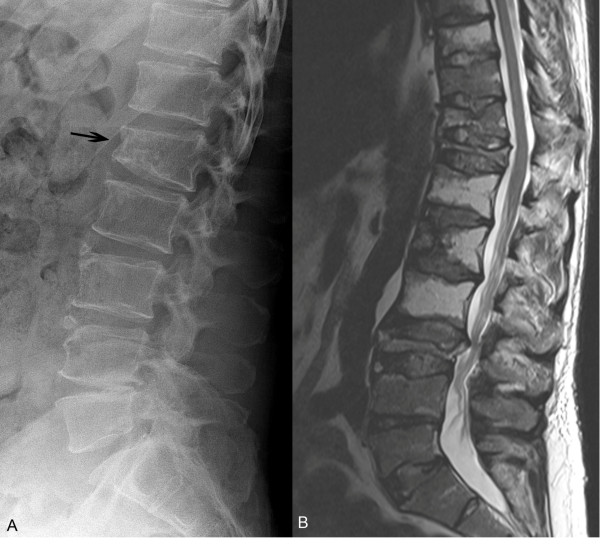
**Multiple Myeloma. A.** A lateral radiograph of the lumbar spine shows diffuse osteopenia and a pathologic compression fracture of the L1 vertebral body (arrow). **B.** A midsagittal T2-weighted MR image of the same patient obtained several months later reveals extensive marrow infiltration (low signal intensity within the marrow of multiple vertebral bodies) and numerous vertebral body compression fractures. Significant central canal stenosis has resulted from retropulsion of vertebral fragments from the pathologic fractures. Radiographs in patients with early multiple myeloma may appear normal or may exhibit only osteopenia resembling senile osteoporosis. Nuclear medicine bone scans are also frequently insensitive to multiple myeloma changes often resulting in false negative results. (Images courtesy of Lindsay J. Rowe, Newcastle, Australia).

Myeloma is the most common primary malignant bone tumor and accounts for about 10 % of all hematologic malignancies [84]. Three diagnostic critieria must be present: (a) greater than 10 % atypical marrow plasma cells and/or biopsy-proved plasmacytoma; (b) monoclonal paraprotein, and (c) myeloma-related organ dysfunction. A bone marrow biopsy or aspirates are necessary to confirm the diagnosis [85]. Myeloma typically infiltrates active red marrow tissue and destroys bone. Typical sites of involvement include the skull, spine, pelvis, ribs, humerus and femur. Initially, radiographs frequently appear normal. Later on, osteoclast stimulation and osteoblast suppression result in diffuse osteopenia which may be difficult to differentiate from senile osteoporosis. With further disease progression, multiple well-circumscribed radiolucencies predominate. Multislice helical axial CT with coronal and sagittal reconstructions is more sensitive than radiographs. (Figure 6) Osteoblastic lesions are extremely rare in myeloma. MRI is more useful than radiography or CT for staging the disease and detecting various patterns of marrow infiltration. FDG-PET can be used to detect multiple myeloma with good sensitivity and specificity. Its ability to assess metabolic activity can be useful, especially when evaluating treatment response and monitoring relapse [84]**.** Additionally, FDG-PET has been shown superior to conventional radiography but less so compared with MRI [86]. In myeloma patients, NM bone scans may show photopenic areas or a negative scan resulting in false negative interpretations [87].

#### Skeletal metastases

**Figure 7:**
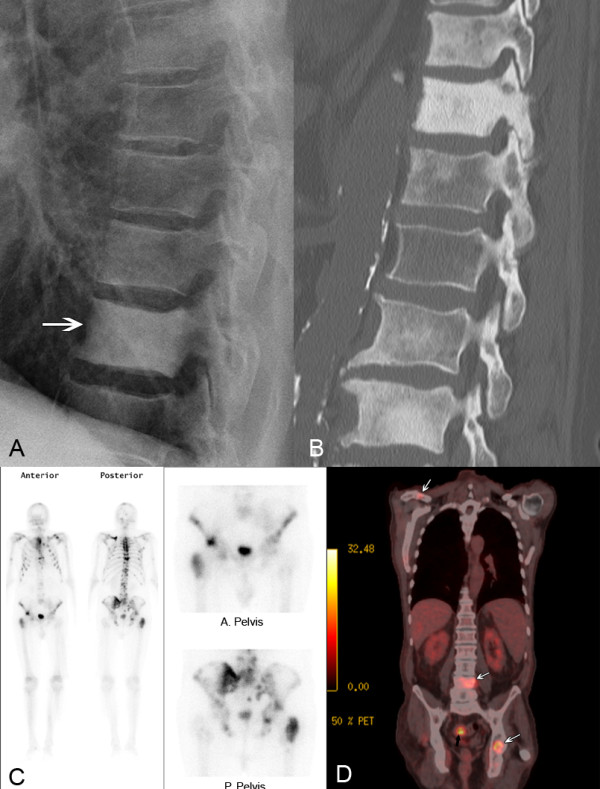
**Skeletal Metastasis. A.** A conventional radiograph shows osteoblastic deposition within the vertebral bodies of this 62 year old man with metastasis from prostate carcinoma (arrow). **B.** A sagittal CT image in another patient reveals extensive osteoblastic involvement throughout the lumbar and thoracic spine in addition to aortic atherosclerosis. **C.** A nuclear medicine planar bone scan in another patient with prostate carcinoma shows multiple areas of increased uptake within the bones of the axial skeleton. Bone scans are the most widely used primary imaging examination for detecting osseous metastasis. Because bone scans are non-specific, areas of abnormality should be followed up with radiographs, CT or MRI to specifically confirm the pathology. **D.** In another patient with colorectal carcinoma, a FDG-PET, fluorine-18-2-fluoro-2-deoxy-D-glucose positron emission tomography fused with CT shows intense uptake at the site of primary bowel carcinoma (black arrow) as well as several sites of osseous metastasis in the left acetabulum, right clavicle, and L4 vertebral body (white arrows). (Images courtesy of Lindsay J. Rowe, Newcastle, Australia).

Metastasis of cancer to the bones is the most common malignant process of the skeleton. More than 80 % of adult cases originate from primary carcinoma of the prostate, breast, lung and bronchus, thyroid, and kidney. Skeletal metastasis is 25 to 30 times more common than any primary bone tumor and as many as 140,000 new cases are identified in the United States annually. Most cases result in osteolytic bone destruction, but some cases are purely osteoblastic or a combination of osteolytic/osteoblastic involvement [84]. The ideal imaging technique for initial staging and monitoring should quickly and accurately identify all active sites of the disease, but no single imaging modality satisfies all the criteria in different situations. MRI, CT, NM bone scan, FDG-PET, and PET/CT are all useful, and any of these may be the best study for an individual patient, depending on their unique clinical circumstances [84]. (Figure 7)

In 2009, the American College of Radiology (ACR) updated their appropriateness criteria for imaging of metastatic bone disease. The ACR reviewed published meta-analyses and systematic reviews with evidence tables focusing on the utility of imaging examinations in differential diagnosis. In summary, they concluded that NM bone scanning is the most widely used primary imaging examination for detecting osseous metastasis. NM is sensitive in detecting osseous abnormalities, but it is nonspecific. Therefore, after an abnormality has been detected, radiographs should be obtained to make sure the abnormality does not represent a benign process. If radiography is not diagnostic, additional lesion workup with MRI, CT, SPECT, or FDG-PET/CT is highly variable and should be based on the clinical situation and lesion location [87]. (Figure 7)

Conventional radiography has low sensitivity for bone destruction and may result in false negative interpretations in cases of skeletal metastasis. It is for this reason that some current guidelines emphasize the use of advance imaging of the spine instead of radiographs to make or to exclude the diagnosis of spinal metastases [88]. Medical referral is recommended if primary contact practitioners do not have immediate access to advanced imaging techniques such as MR imaging, CT, and NM bone scanning studies. In geographical areas with limited access to primary care physicians, it some authorities argue that it is reasonable to include conventional radiography (with or without erythrocyte sedimentation rate) in the evaluation of persons with red flag indicators of suspected skeletal metastasis [55].

#### Metastatic spinal cord compression (MSCC)

MSCC represents compression of the dural sac and its contents—the spinal cord and cauda equina—by an extradural mass. Metastatic lung, breast and prostate cancers are the commonest malignancies causing MSCC and account for over 50 % of cases [89]. In 7 % of patients the site of primary tumour may remain unidentified [90]. In 23 % of patients, MSCC will be the first presenting problem. Because patients with known malignancy may also have spinal cord compression from a non-malignant cause, it is important to differentiate MSCC from other causes such as degenerative stenosis and osteoporotic compression fractures [91]. According to recommendations in 2008 by the National Collaborating Center for Cancer, every cancer network should ensure that there is local access to urgent magnetic resonance imaging (MRI) within 24 hours for all patients with suspected MSCC. This service should be available outside normal working hours and with 24-hour capability in centres treating patients with MSCC [91]. More specific Selected Imaging Recommendations from 2008 NICE Guidelines on MSCC [88] are as follows.

MRI of the spine in patients with suspected MSCC should be supervised and reported by a radiologist and should include sagittal T1 and/or short T1 inversion recovery (STIR) sequences of the whole spine, to prove or exclude the presence of spinal metastases. Sagittal T2 weighted sequences should also be performed to show the level and degree of compression of the cord or cauda equina by a soft tissue mass and to detect lesions within the cord itself. Supplementary axial imaging should be performed through any significant abnormality noted on the sagittal scan.

Consider targeted CT scan with three-plane reconstruction to assess spinal stability and plan vertebroplasty, kyphoplasty or spinal surgery in patients with MSCC.

Consider myelography if other imaging modalities are contraindicated or inadequate.

Myelography should only be undertaken at a neuroscience or spinal surgical centre because of the technical expertise required and because patients with MSCC may deteriorate following myelography and require urgent decompression.

Do not perform plain radiographs of the spine either to make or to exclude the diagnosis of spinal metastases or MSCC.

In patients with a previous diagnosis of malignancy, routine imaging of the spine is not recommended if they are asymptomatic. (Serial imaging of the spine in asymptomatic patients with cancer who are at high risk of developing spinal metastases should only be performed as part of a randomised controlled trial.)

Perform MRI of the whole spine in patients with suspected MSCC, unless there is a specific contraindication. This should be done in time to allow definitive treatment to be planned within 1 week of the suspected diagnosis in the case of spinal pain suggestive of spinal metastases, and within 24 hours in the case of spinal pain suggestive of spinal metastases and neurological symptoms or signs suggestive of MSCC, and occasionally sooner if there is a pressing clinical need for emergency surgery.

#### Cervical spine myelopathy

Causes of cord compression include trauma, tumors, infection, vascular disease, degenerative conditions, demyelinating disorders, spinal stenosis, & central cervical disc herniation [52]. Clinicians should be aware that nearly all of the clinical tests for cervical spine myelopathy are poor screening tools, which implies that manually oriented clinicians may mistakenly proceed with treatment when it is not indicated. [92]. Patients presenting with signs and symptoms of cervical spine myelopathy should therefore undergo appropriate investigation before proceeding with manual therapy interventions. Cervical spine radiographs including oblique projections are indicated in patients with suspected cervical compressive myelopathy or radiculo-myelopathy. MRI should also be performed to identify cord compression and/or high signal intensity intramedullary cord lesions, the latter of which are associated with a poorer prognosis even after decompressive surgery. If MRI is unavailable, CT-myelography should be considered. In addition to imaging, electrophysiologic testing such as somatosensory evoked potentials (SSEP) may be useful [52,93].

#### Suspected atlantoaxial instability (AAI)

AAI is of particular importance to chiropractors and other clinicians involved in manual therapy of the cervical spine. Many conditions result in osseous abnormalities such as nonunion or agenesis of the odontoid, rupture, laxity, or absence of the transverse ligament, or other upper cervical spine pathologies. These include but are not limited to: a) active inflammatory arthritis such as rheumatoid arthritis (RA), psoriatic arthropathy, ankylosing spondylitis, and systemic lupus erythematosus; b) Congenital disorders and hereditary connective tissues disorders such as spondyloepiphyseal dysplasia, os odontoideum, and several syndromes including Klippel-Feil, Morquio, Down (20 % of Trisomy 21 patients are born without a transverse ligament), Ehlers-Danlos type III, and Marfan; c) traumatic conditions such as C1 or C2 fracture or dislocation. Lateral radiographs of the cervical spine obtained in flexion and extension are indicated in suspected AAI, however, a single lateral cervical radiograph with the patient in supervised comfortable flexion should reveal any subluxation in patients with suspected instability. In adults, the atlantodental interval should not exceed 3 mm in neutral, flexion, or extension neck positions. In the presence of neurologic signs and symptoms, MRI or CT are indicated to reveal osseous abnormalities, stenosis, and spinal cord lesions [52,94].

### Costs of spine imaging

The skyrocketing costs of imaging for LBP and NP in older adults have been attributed to a number of factors. One significant factor is the dramatic overall increase in elderly persons. Between 1991 and 2002 in the US, for example, there was a 42.5 % increase in the number of Medicare beneficiaries [30]. According to the American Association of Retired Persons (AARP), in 2007, there were 44 million people on Medicare in the US, and that number is expected to increase by 80 % to 79 million by 2030 [95]. Similarly, among US Medicare beneficiaries between 1991 and 2002, there was a 131 % increase in LBP patients and a 387 % increase in charges for LBP evaluation and management [30]. One 2009 study of imaging for acute LBP in over 35,000 US Medicare patients revealed that 28.8 % of beneficiaries were imaged within the first 28 days of the onset of pain and an additional 4.6 % were imaged between 28–180 days. Of the imaged patients, 88.2 % had radiographs and 11.8 % had CT or MRI as their initial study [29]. Evidence suggests that many radiography, CT and MRI studies are ordered unnecessarily in patients with simple mechanical back pain and no red flags for serious disease [30]. It has been estimated that in LBP patients, overutilization of conventional radiography occurs in as many as 26 % of cases and of MRI and CT in 66 % of cases [96].

A recent survey of Australian chiropractors set out to determine how chiropractors manage people with acute LBP and to determine whether this management is in accordance with recommendations from an evidence-based acute LBP guideline. One recommendation was directed at minimising the use of conventional radiographs. The authors presented four clinical vignettes of patients who, according to the guideline, would not require conventional lumbar radiographs, and one vignette of a patient with a suspected vertebral fracture. Of the 274 chiropractors that responded, 95 % indicated that they would (appropriately) obtain radiographs in the patient with a suspected vertebral fracture, whereas 68 % indicated that they would also obtain radiographs in the four patients with whom radiography was not indicated. This study reveals low compliance with recommendations from an evidence-based guideline for acute LBP [97].

In the US, the cost of spine imaging studies in the elderly are born either by Medicare, by additional insurance coverage, or less commonly by the patient. Individual charges for these services vary according to the imaging modality, the anatomic region imaged, the geographic location where the imaging study is performed, and by the type and extent of Medicare or insurance coverage. Furthermore, reimbursements are often considerably less than the amount charged by imaging centers and hospitals. Sample comparisons of typical charges and actual Medicare reimbursements in the US are displayed in Table 4.

**Table 4:** US Medicare Reimbursements for Imaging Studies 2010*

**Examination**		**Medicare National Average Global Fee**		
	**CPT Code**	**$ Submitted**	**$ Allowed**	**% Allowed**
**LUMBAR SPINE**				
Radiographs 2 or 3 views	72100	73.12	23.70	32.4
CT without contrast	72131	329.71	97.11	29.5
CT with contrast	72132	356.98	99.61	27.9
CT with and without contrast	72133	547.05	169.74	31.0
Injection for myelogram	62284	489.58	107.97	22.1
MR without contrast	72148	802.75	230.36	28.7
MR with contrast	72149	736.35	224.10	30.4
MR with and without contrast	72158	1,088.47	302.33	27.8
**CERVICAL SPINE**				
Radiographs 2 or 3 views	72040	68.61	22.56	32.9
CT without contrast	72125	240.39	68.81	28.6
CT with contrast	72126	351.46	98.07	27.9
CT with and without contrast	72127	484.23	153.25	31.6
MR without contrast	72141	772.47	224.40	29.0
MR with contrast	72142	732.39	225.78	30.8
MR with and without contrast	72156	1,049.86	302.71	28.8
**GENERAL**				
DXA Scan Axial	77080	172.88	59.30	34.3
Ultrasound screen for AAA	76770	190.10	69.71	36.7
			Mean % Allowed:	30.0

*https://www.codemap.com/cpt.cfm?cpt_code=72131 Accessed Nov 19, 2011.

Significant global variations in the cost of diagnostic imaging examinations have been identified. The International Federation of Health Plans (IFHP) tracks health care financing and health care delivery costs of 100 member companies in 30 countries. In 2010, the IFHP reported the range of fees for various diagnostic imaging studies including MRI and CT scans of the abdomen, head, and pelvis in several countries [98]. (Table 5) While these selected fees neither specifically address spine imaging nor elderly patients *per se*, they do represent a relative comparison of the costs of CT and MRI in each country. What is most significant is the wide variability of costs within the US alone where the fees for MRI range from $509 to $2,590, and CT ranges from $82 to $1564 (All US$), and represent the highest overall costs of all countries. It should be noted that the US fees displayed in Table 5 are derived from independent databases tracking payment levels by third party payers, and publicly reported sources. These reflect commercially negotiated claims-based, fee-for-service paid charges between payers and providers / hospitals. These fees vary widely by state, by specialty, by hospital and by payer [98]. The fees displayed in Table 4 represent publically funded Medicare payments, principally to elderly patients.

**Table 5:** Comparison of Imaging Fees in Various Countries*

	**MRI**	**CT**		
	**Head**	**Abdomen**	**Head**	**Pelvis**
United Kingdom	$187	$187	$187	$187
Spain	$234	$117	$117	$117
Canada	$304	$61	$65	$98
France	$398	$179	$179	$215
Australia	$439	$487	$234	$386
Chile	$505	$249	$220	$179
New Zealand	$603	$447	$227	$302
Germany	$632	$374	$287	$374
Switzerland	$874	$411	$360	$391
United States (Avg)	$1 009	$536	$464	$487
**All Countries (Avg)**	**$518**	**$304**	**$234**	**$273**
United States (Low)	$509	$164	$82	$142
United States (High)	$2 590	$1 564	$1 430	$1 404

*Adapted from: Sackville T: International Federation of Health Plans, 2010 Comparative Price Report, Medical and Hospital Fees by Country. IFHP Council Meeting, November 2010.

http://www.ifhp.com/documents/IFHP_Price_Report2010ComparativePriceReport29112010.

In Australia, overall diagnostic imaging services for the year 2011totaled 1.15 billion (AU$) of which 12.1 % was for musculoskeletal DUS, 58.1 % for spine CT, 19.4 % for NM bone scanning, 6.2 % for spine radiography, and 4.3 % for MRI of the spine [99].

### Ionizing radiation exposure associated with spinal imaging

Radiation exposure from diagnostic imaging represents a major source of artificial ionizing radiation that accounts for a significant proportion of the collective dose received by the population. With the exception of MRI and DUS, diagnostic imaging of the spine poses significant risk because it involves the irradiation of large exposure fields that include multiple radiosensitive organs. These comparatively large doses contribute to the lifetime risk of radiation-induced carcinogenesis. Studies by Simpson [100], Richards [101] and others [102] have estimated the relative effective doses (mSv) of spine radiography, CT, PET, and bone density studies. These effective doses, along with estimates of associated cancer risk, are summarized and compared to the doses associated with chest radiography in Table 6. It must be emphasized, however, that any such estimations vary according to a wide range of imaging parameters that are employed at different institutions. The estimations also vary considerably according to patient size and tissue thickness and the imaging modality employed. Conventional radiographs, DXA scans, and Quantitative CT (QCT) studies result in much less radiation exposure than CT, NM, and FDG-PET scans. (Table 6)

**Table 6:** Radiation Dose and Estimated Cancer Risk Associated with Selected Spine Imaging Studies [87,100-102]

						**Estimated Cancer Risk**	
**Imaging Modality**	**Region**	**Study**	Relative Radiation Level*	**Estimated Effective Dose (mSv)**	**Equivalent # of chest x-rays**	**Risk coefficient (X10-4)**	**Risk Ratio**
**Radiography**							
	Chest	PA view	Minimal	0.02	1	0.01	1 in 1,000,000
		Lat view	Minimal	0.02	1	0.01	1 in 1,000,000
	Cervical	AP view	Minimal	0.12	6	0.06	1 in 180,000
		Lat view	Minimal	0.02	1	0.01	1 in 1,000,000
	Lumbar	AP view	Medium	2.2	110	1.1	1 in 9,000
		Lat view	Medium	1.5	75	0.75	1 in 10,500
**CT**							
	Thoracic	Whole T-spine	High	10	500	5.5	1 in 1,800
	Lumbar	Whole L-spine	Medium	5.6	280	3.1	1 in 3,200
**Tc-99 m bone scan**							
		Whole body	Medium	3.5	175	1.9	1 in 5,200
**FDG-PET**							
		Whole Body	Medium	7	350	3.8	1 in 2,500
**Bone density**							
	QCT	L-spine	Minimal	0.1	5	0.05	1 in 200,000
	DXA	L-spine	Minimal	0.005	0.25	0.025	1 in 4,000,000
**MRI**							
			None	0	0	0	0
**Ultrasound**							
	Abdominal	Aorta	None	0	0	0	0

* Relative Radiation Level: 0 mSv = None; <0.1-1 mSv = Minimal; 1–10 mSv = Medium; 10–100 = High.

Limiting CT of the spine to the smallest area necessary to answer the clinical question has a dramatic effect on the estimated cancer risk for individual patients. Cancer risks are summative, so spine CT imaging needs to be considered in light of the total radiation risk to the patient over their lifetime [101].

Evidence-based management of spine disorders in the elderly population has received little research attention. Furthermore, methods for developing recommendations about diagnostic tests are far from completely explored [103]. Future direction should aim to develop diagnostic imaging recommendations intended to optimize care in the elderly that are informed by systematic reviews and an assessment of the benefits, harms, and costs of available options.

A summary of key points of the role of diagnostic imaging for spinal disorders in the elderly follows [59,65]:

Arthritis and degenerative disc disease are highly prevalent in the elderly population.

While prevalence of serious pathologies remains low, they increase with age. Be alert to red flags of cancer, infection, cauda equina syndrome, and presence of severe or progressive neurologic deficits (multiple levels).

Immediate imaging and/or referral are indicated if major red flags are present.

Refrain from routine, immediate lumbar spine imaging in adult patients with acute or subacute low-back pain and without red flags suggesting a serious underlying pathology.

CT and MRI offer better characterization of most musculoskeletal diseases than conventional radiography with the exception of suspected fracture or arthritis.

CT should be employed for suspected osseous lesions or occult fractures.

MRI should be employed for suspected soft tissue masses or invasion.

Consider MRI when a diagnosis of spinal malignancy, infection, fracture, cauda equina syndrome or ankylosing spondylitis or another inflammatory disorder is suspected.

Only offer an MRI scan for non-specific low back pain within the context of a referral for an opinion on spinal surgery.

A subspecialty radiologist’s interpretation is necessary to provide the greatest amount of useful clinical information.

## Conclusions

This narrative review of imaging for spine disorders aims to assist clinicians in their clinical decision making with elderly patients. A more conservative approach to the diagnostic evaluation is advisable, both from a health risk perspective and from a resource control perspective. While age over 65 may appear to be a reasonable age cut-off to justify ordering imaging studies, symptom duration alone is not. Lumbar radiographs and evaluation of erythrocyte sedimentation as an initial assessment of patients with minor risk factors for cancer (unexplained weight loss or age > 50 years) is a reasonable approach for LBP patients. When available, MRI is the preferred modality if a diagnosis of spinal malignancy, infection, fracture or inflammatory disorder is suspected, where CT scan is used for suspected bony lesions or occult fractures.

## Abbreviations

AAA, abdominal aortic aneurysm; AAI, atlantoaxial instability; AARP, American Association of Retired Persons; ACR, American College of Radiology; BMD, bone mineral density; CCSR, Canadian cervical spine rule for radiography in alert and stable trauma patients; CES, cauda equina syndrome; CPT code, current procedural terminology code; CT, computed tomography; DLS, degenerative lumbar spondylolisthesis; DLSS, degenerative lumbar spinal stenosis; DUS, diagnostic ultrasound study; DXA, dual energy x-ray absorptiometry; ESR, erythrocyte sedimentation rate; FDG-PET, fluorine-18-2-fluoro-2-deoxy-D-glucose positron emission tomography; FRAX®, fracture risk assessment tool (World Health Organization); IFHP, International Federation of Health Plans; MRI, magnetic resonance imaging; MSCC, metastatic spinal canal compression; mSv, millisievert; NASS, North American Spine Society; NICE, National Institute for Health and Clinical Excellence; NM, nuclear medicine bone scan; PET, positron emission tomography; PET/CT, positron emission tomography combined with computed tomography; PSA, prostate specific antigen; QCT, quantitative computed tomography; RA, rheumatoid arthritis; SPECT, single photon emission computed tomography; SSEP, somatosensory evoked potentials; TFNP, Bone and Joint Decade 2000-2010 task force on neck pain and its associated disorders; US, United States; USPSTF, US Preventive Services Task Force.
